# A report of the complete mitochondrial genome of *Bisetocreagris titanium* (Arachnida: Pseudoscorpiones: Neobisiidae) from Yunnan Province, China

**DOI:** 10.1080/23802359.2021.1987164

**Published:** 2021-10-19

**Authors:** Huifeng Zhao, Haifeng Chen, Yunchun Li

**Affiliations:** a Hebei Key Laboratory of Animal Diversity, College of Life Science, Langfang Normal University, Langfang, Hebei, China; b College of Life Science, China West Normal University, Nanchong, Sichuan, China

**Keywords:** Arachnida, mitochondrial genomes, phylogenetic relationship, Pseudoscorpiones

## Abstract

A complete mitogenome of a cave dwelling pseudoscorpion *Bisetocreagris titanium* is reported here. The mitogenome is a circular DNA molecule with a length of 14,756 base pairs (bp), and it contains 13 protein coding genes (PCGs), 22 transfer RNAs (tRNAs), 2 ribosomal RNAs (rRNAs), and 1 putative control region. Phylogenetic analysis of 30 Arachnida species was performed based on the amino acid datasets of 13 PCGs, and the result indicated Pseudoscorpiones is the sister lineage of Acariformes. This result is congruent with the former phylogenetic results of mitogenomes, but incongruent with the results of morphological characters and/or ribosomal DNA data that indicated Pseudoscorpiones are positioned in a clade with the Solifugae.

Pseudoscorpiones resemble scorpions in shape. They lack a tail-shaped posterior abdomen and contain a tail section with a venomous needle (Weygoldt 1969). The body size of Pseudoscorpiones ranges from 1 to 8 millimeters, and they inhabitat a variety of environments, such as leaf litter, caves, *etc*. *Bisetocreagris* Ćurčić, 1983 is a Pseudoscorpiones classified in the Neobisiidae, and the genus only occurs in China. Most species in *Bisetocreagris* are cave dwellers, including *B. taitianium* (Mahnert 2003) (Mahnert 2003). The type locality of *B. taitianium* is the Xiaoguoquan Cave, Wude Town, Zhenxiong County, Zhaotong Prefecture, Yunnan Province, China. The eyes of *B. taitanium* are vestigial and adapted for the dark cave envirnoment. The family Neobisiidae has not been analyzed using DNA evidence. Here we contribute to the bioinformatics and molecular systematics of Pseudoscorpions by analyzing *B. titanium* from the type locality (25.586027°N, 104.762101°E) from a specimen collected on 30.VIII.2020. The specimen was deposited in the Museum of China West Normal University (voucher no. ps.YN000352, Prof. Ai-Min Shi, aiminshi2003@126.com). The Genbank accession number of this mitogenome is MZ029090.

This mitogenome represents the first in the Neobisiidae and the third for the order Pseudoscorpiones (Ovchinnikov and Masta 2012). The mitogenome of *B. titanium* is a typical circular DNA with a length of 14,756 bp and an AT frequency of 73.98% and GC frequency of 26.02%. The AT skew is −0.0501, and GC skew is −0.1102. The mitogenome contains 37 genes, including 13 protein coding genes (PCGs), 22 transfer RNAs (tRNAs), and 2 ribosomal RNAs (rRNAs), as well as a putative control region. The start codons of the 12 PCGs are ATD, and for ND3 it is TTG. The stop codons for 11 PCGs are TAR, and for ND2 and ND5 they are terminated with an incomplete stop codon T–. Most tRNAs are short and do not form the typical cloverleaf structure. They are similar to the tRNAs in the mitogenomes of other published Pseudoscorpiones (Ovchinnikov and Masta 2012) and other organisms classified in the Arachnida (Pons et al. 2019). The tRNA details are: tRNA^Gln^ and tRNA^Thr^ lack of DHU and TΨC arms; tRNA^Ser1^ lacks of DHU arm; nine tRNAs, i.e., tRNA^Ala^, tRNA^Asp^, tRNA^Glu^, tRNA^Gly^, tRNA^Pro^, tRNA^Ser2^, tRNA^Val^, tRNA^Trp^, tRNA^Tyr^ lack of TΨC arm; the remaining 10 tRNAs, i.e., tRNA^Arg^, tRNA^Asn^, tRNA^Cys^, tRNA^His^, tRNA^Ile^, tRNA^Lys^, tRNA^Leu1^, tRNA^Leu2^, tRNA^Met^ and tRNA^Phe^ can encode to the regular cloverleaf structure. The control region of *B. taitanium* is 805 bp in length and does not contain a tandem repeat region.

Phylogenetic analysis of the *B. taitanium* mitogenome with 29 other Arachnia ingroups and one outgroup was performed using the maximum likelihood optimality criterion in IQTREE (Nguyen et al. 2015) with the model of MtZoa (Rota-Stabelli et al. 2009) and 1000 regular bootstrap replicates (-m MtZoa + F + I + G4 -b 1000). The analysis fully resolved *B. taitanium* in a clade with *Paratemnoides elongatus*, which is classified in the family Atemnidae in the Pseudoscorpiones (Figure 1). Furthermore, this result showed that the Pseudoscorpiones is the sister lineage to the Acariformes. This result is consistent with former mitogenomic phylogenetic analyses (Ovchinnikov and Masta 2012; Liu et al. 2015) (Figure 1), but incongruent with the results of morphological and/or ribosomal sequence data that indicated Pseudoscorpiones is a clade within the Solifugae (Wheeler and Hayashi 1998; Giribet et al. 2002; Weygoldt and Paulus 2009).

**Figure 1.**
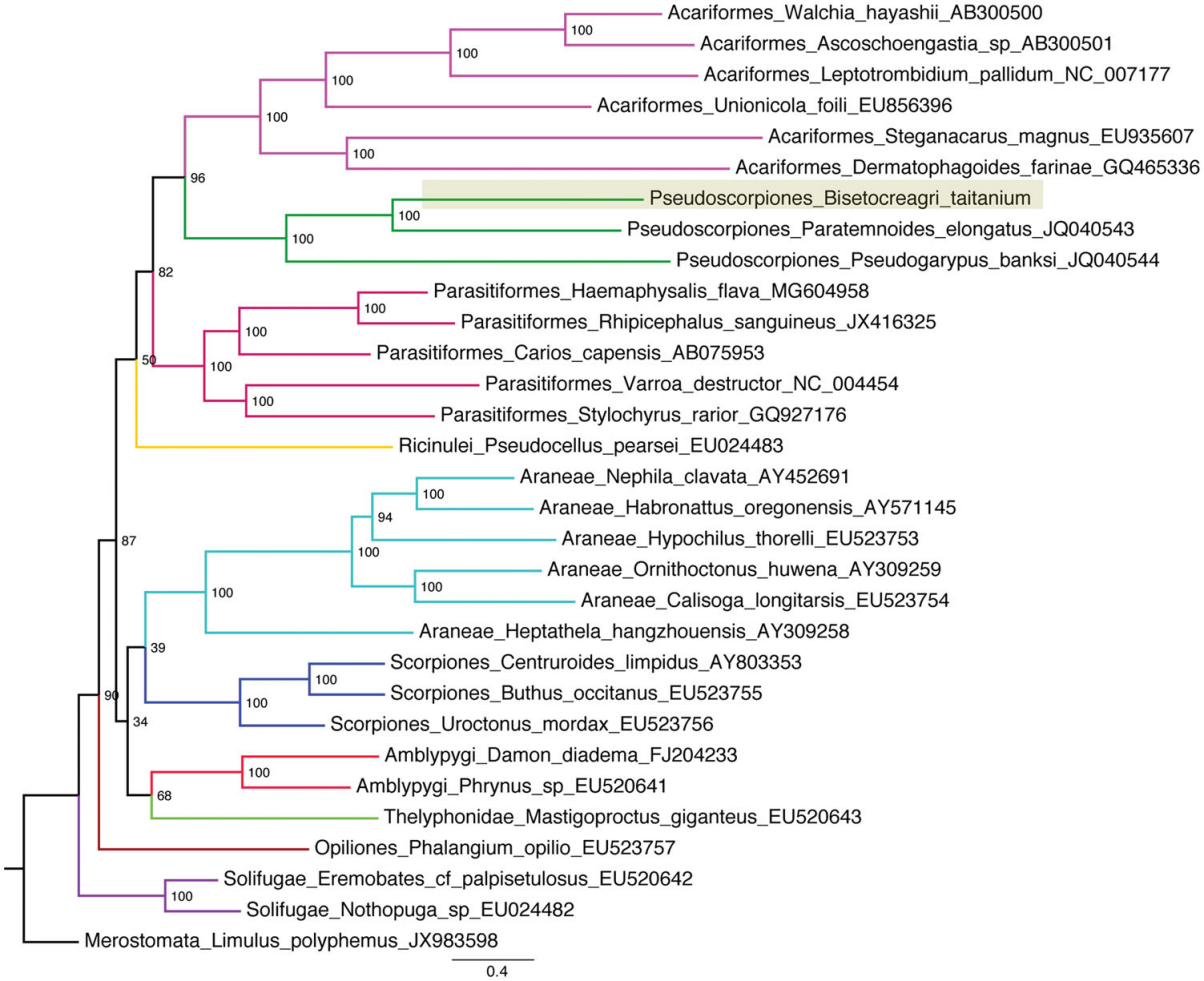
The ML phylogenetic tree constructed based on the amino acid dataset of mitogenomes in Arachnida. *Bisetocreagris taitanium* clade was highlighted. Numbers aside the nodes are the support values based on 1,000 bootstrap replicates.

## Data Availability

The data that support the findings of this study are openly available in the NCBI Genbank database at https://www.ncbi.nlm.nih.gov, reference number MZ029090. The associated BioProject, SRA, and Bio-Sample numbers are PRJNA730386, SRS9008569, and SAMN19229830, respectively.
